# Segtor: Rapid Annotation of Genomic Coordinates and Single Nucleotide Variations Using Segment Trees

**DOI:** 10.1371/journal.pone.0026715

**Published:** 2011-11-01

**Authors:** Gabriel Renaud, Pedro Neves, Edson Luiz Folador, Carlos Gil Ferreira, Fabio Passetti

**Affiliations:** 1 Bioinformatics Unit, Clinical Research Coordination, Instituto Nacional de Cancer (INCA), Centro, Rio de Janeiro, Brazil; 2 Clinical Research Coordination, Instituto Nacional de Cancer (INCA), Centro, Rio de Janeiro, Brazil; University of North Carolina at Charlotte, United States of America

## Abstract

Various research projects often involve determining the relative position of genomic coordinates, intervals, single nucleotide variations (SNVs), insertions, deletions and translocations with respect to genes and their potential impact on protein translation. Due to the tremendous increase in throughput brought by the use of next-generation sequencing, investigators are routinely faced with the need to annotate very large datasets. We present Segtor, a tool to annotate large sets of genomic coordinates, intervals, SNVs, indels and translocations. Our tool uses segment trees built using the start and end coordinates of the genomic features the user wishes to use instead of storing them in a database management system. The software also produces annotation statistics to allow users to visualize how many coordinates were found within various portions of genes. Our system currently can be made to work with any species available on the UCSC Genome Browser. Segtor is a suitable tool for groups, especially those with limited access to programmers or with interest to analyze large amounts of individual genomes, who wish to determine the relative position of very large sets of mapped reads and subsequently annotate observed mutations between the reads and the reference. Segtor (http://lbbc.inca.gov.br/segtor/) is an open-source tool that can be freely downloaded for non-profit use. We also provide a web interface for testing purposes.

## Introduction

The advent of next-generation sequencing (NGS) technologies has enabled a drastic growth in the number of sequencing projects by largely increasing the sequence output and by lowering overall costs. Certain projects involve the sequencing of an organism whose genome is already available. These projects, called resequencing projects, generally involve two steps: the mapping of reads onto the known genome and the subsequent analysis of divergent features between the reference genome and the mapped sequences[1]. These projects can be readily used to identify mutations in the sample, characterize gene expression (RNA-Seq), identify various genomic features like protein binding sites (ChIP-Seq) or pinpoint the location of microRNAs (see [2] for a discussion of the various aspects).

Once the mapping is completed, investigators are often left with the daunting task of identifying the relative position of a large number of single nucleotide variations (SNVs), insertions, deletions and genomic translocations to existing genomic features. Recent attention has also been given to the task of representing and handling these features [3]. The annotation of translocations can be especially useful for cancer studies[4]. Among the genomic features that investigators might be interested in are high-quality mRNAs, ESTs or gene predictions already mapped to the reference sequence. Another task might involve the identification of which genomic coordinates or genomic intervals overlap or span known genomic features (see [5] for an example). This is required to gather statistics regarding the genomic context of the mapped reads or to calculate enrichment. For instance, knowing the percentage of reads that map to a known exon for an exome sequencing protocol is instrumental in evaluating the efficiency of the method. Furthermore, research groups are sometimes faced with the challenge of identifying the closest transcription start site (TSS) for various genomic coordinates (see [6] for an example). All these problems basically amount to the task of finding which genes overlap a given genomic coordinate or interval.

Traditionally, research groups have solved the problem of querying known gene annotations by either querying remote public databases or by using a local Database Management System (DBMS) such as MySQL, Oracle or PostgreSQL to store the genomic features they wish to use as source of annotation. The former has the advantage of being up-to-date but cannot be rapidly queried for very large sets. Although some work has been done to improve query time in DBMS[7], the latter still creates systems that cannot be easily carried over to other labs and require someone with DBMS expertise to create, optimize and update the data tables. As NGS technologies become increasingly common and may play a crucial role in the rise of personnalized medicine[8], the need for standalone software tools becomes ever more apparent.

We introduce Segtor, a rapid annotation tool aimed at NGS studies for genomic coordinates, intervals, SNVs, insertions, deletions and translocations. It allows users to determine which are either upstream, downstream and within exons or introns. For genomic intervals, it can assess which are spanning either exons, introns, both exonic and intronic regions or lying upstream or downstream of genes. Segtor can determine which SNVs are synonymous or non-synonymous and which landed in intronic regions in the vicinity of a splice acceptor or donor site. It also allows detection of the closest TSS for a set of genomic coordinates. Our method is designed to run without the need for a DBMS and uses instead segment trees[9] to store the boundaries of the genes to rapidly retrieve which genes overlap a coordinate or an interval. A comparison between Segtor and other tools aiming at tackling similar problems can be found in the discussion. Segtor distinguished itself from currently available software by providing ample information regarding the various splicing isoforms in the vicinity of a SNV, readily producing the altered protein sequence, displaying useful annotation statistics and providing a holistic ad hoc tool for various annotation needs for any species available on the UCSC Genome Browser.

## Methods

**Figure 1:**
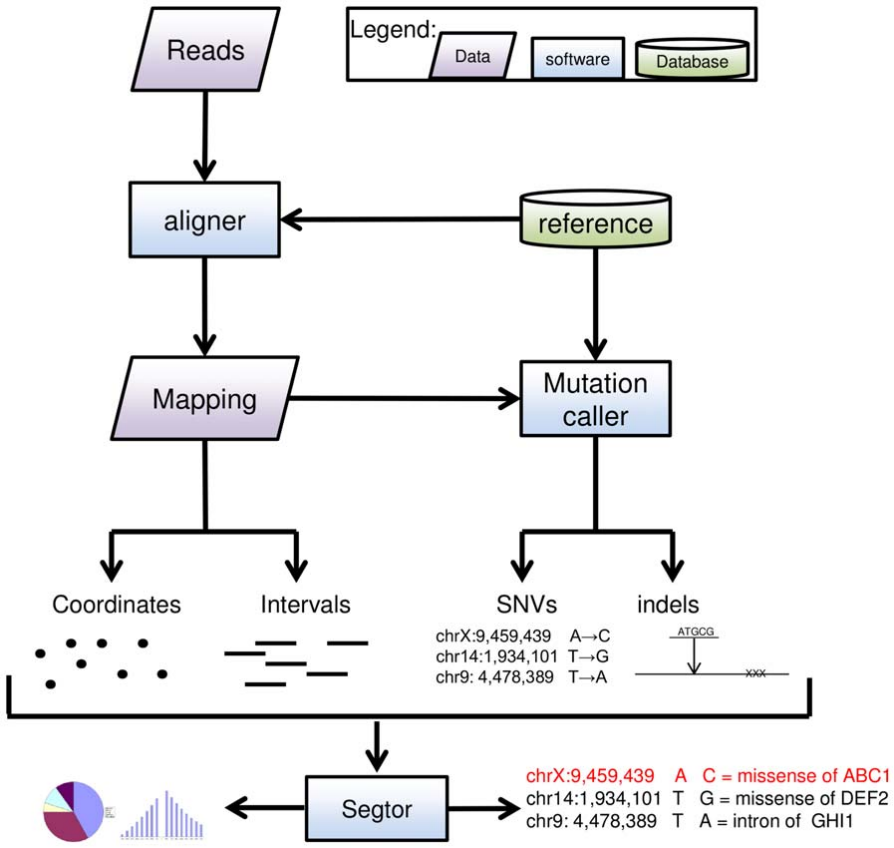
A diagram representing a possible re-sequencing workflow to illustrate the usefulness of Segtor in obtaining statistics on the position of reads with respect to known genes and evaluating the impact of SNVs and indels on protein translation. Reads are mapped using an aligner to the reference, the 5′ ends and genomic intervals of the mapped reads can be used as input for Segtor to determine how many landed within various portions of genes. Furthermore, Segtor can measure the distance to the closest transcription start site. The SNVs and indels that were detected can be also classified using Segtor into which SNVs land within coding portions and which potentially affect the resulting protein sequence.

The software can be downloaded and used locally for large datasets or accessed through a user-friendly web interface for small queries. Figure 1 presents how Segtor can be instrumental within an NGS workflow.

### Running a local version

Upon using the local version of Segtor, users have the ability to select any species whose genome is available on the UCSC Genome Browser. The software currently supports the following gene databases: RefSeq [10], Ensembl Genes[11], UCSC Known Genes[12] and dbEST[13]. When annotating SNVs, Segtor can report which ones have already been reported in dbSNP[14]. Segtor can also report the mutated protein sequence, useful for for proteomic studies, when running against SNVs, indels and translocations. Please note that we provide a putative protein sequence with the modifications caused by the mutations and do not try to predict the influence on splice patterns (see [15] or [16] for examples of programs aimed at predicting splice patterns due to SNVs).

The software requires an archived index of the segment tree for the database and species the user wishes to use. Some indices are available on our website and, if the user wishes to build new ones, Segtor will automatically connect to the UCSC FTP site and download the genome along with the database files for the genes. It will proceed to build a segment tree using the database files containing the annotation data and the genome for extracting the exonic sequences and store the resulting data structure as an archived index on the local hard drive for later use. The user has the possibility of choosing where to store these files. Once the segment tree is loaded into memory, it can be used to rapidly annotate large sets of data due its speed. If the database files are obsolete, the user can instruct Segtor to retrieve newer database files.

The local version needs to be launched directly from the command line of a Linux or MacOS terminal. The user must specify the input files, the species assembly, the gene database, the range at which to consider genes as well as the annotation mode to launch. The user can choose from the following 5 modes:

1. Annotate genomic coordinates with respect to genes. This mode will report which coordinates are located within the exons, introns of a gene or which are upstream or downstream within a certain range. The tally will detail how many coordinates fell within each category to provide an overall view. It will also report the indices of the exons and the introns within which the coordinates landed.
2. Characterize the position of genomic intervals of less than 2kb with respect to genes. This option can be used to measure how many reads fall within which genes and what are their relative position (contained within an exon, spanning exonic and intronic regions, etc). The tally reports how many intervals were classified within each category.
3. Determine the relative position of SNVs to genes and which are non-synonymous versus synonymous. This mode also includes the possibility of identifying which were already described in dbSNP. Upon reporting the results for a SNV, the program classifies the overlapped genes according to their potential impact on the protein sequence going from a SNV landing in an exon causing non-synonymous change being ranked the highest to one occurring downstream being the lowest. A tally is produced as to how many SNVs were labeled as a non-synonymous or synonymous mutation and how many landed within various parts of genes.
4. Determine the closest TSS to a set of genomic coordinates regardless of strand. The user can provide bin sizes and number of bins to create histograms of the distance to TSSs for publication.
5. Annotate insertions, deletions and translocations with respect to genes and produce a putative protein sequence resulting from the ones landing in exonic regions. No tally is performed for this mode.

To allow investigators to examine the results according to the specific biological questions being asked, Segtor produces 3 different files, one reporting all the transcripts, another reporting the most relevant transcript on a per gene basis and another reporting the most relevant transcript overall. The most relevant transcript means that we prioritize transcripts according to the position of the feature with respect to them. For instance, if a coordinate lands within the exon of one transcript and within the intron of another, the former will be reported over the latter.

Users also have the option of creating their custom databases using files in .psl or .bed format describing the genomic feature they wish to use. This can be instrumental in discovering not only which reads overlap an known genomic feature but also to identify novel functional elements of the genome by identifying which reads do not overlap a known feature. A script is provided to allow conversion from the BAM format[17], BED format, the output from SNVMix[18] (for SNVs) and VarScan[19] (for insertions/deletions) to the native input format used by Segtor.

#### The data structure

**Figure 2:**
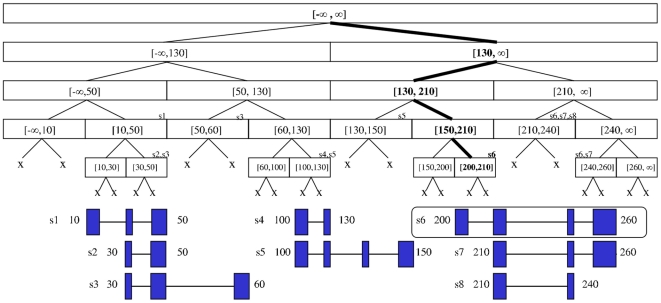
Example of a segment tree holding data from 8 different transcripts belonging to 3 different genes. The nodes are marked with the interval they represent. The transcripts contained in the nodes are located above the top right corner of the node. The path in bold represents a search performed to find transcripts overlapping coordinate 204 leading us to find s6.

Segtor determines which genes are overlapped by a given coordinate or interval by storing gene data in segment trees according to their position on the chromosome in an approach similar to the binning approach used by UCSC and recursively searching them. For a given genomic bin, if the amount of features contained therein exceeds a certain threshold, the software stores the element in a segment tree rather than a simple array to increase speed. A segment tree is a balanced binary tree built using the unique endpoints of the genomic feature the user wishes to query (an example of a segment tree built using genomic annotation data can be seen in figure 2). The internal nodes of the tree represent an interval on a chromosome and hold a reference to the genes that span it. To maximize efficiency, trees are built once for a given species/database pair and stored on the disk as an index for faster retrieval in subsequent uses. As mentioned earlier, the user has the option of downloading indices on our website however, to use a different species or database, the user can create their own index.

#### Annotation

The software proceeds to read the input files containing the genomic coordinates, intervals or SNVs to annotate, computes the appropriate genomic bins to search and queries the corresponding segment trees. The search algorithm in the tree proceeds slightly differently whether the input is a simple coordinate or a genomic range.

For simple coordinate, SNVs or deletions, it will launch a recursive search in the segment tree starting at the root and called recursively on the child whose interval contains the coordinate. At each call, the transcripts or genomic features stored in the nodes are returned and finally stops upon reaching a leaf node.

For genomic intervals, insertions or other queries involving the use of a genomic range, the program computes both the lowest and highest coordinates of the genomic span to consider. A recursive search is then performed on a child node if its interval spans both the lowest coordinate and the highest one. However, if the range is large enough, we will eventually reach a non-leaf node whose left child spans the lowest coordinate and whose right child spans the highest coordinate in which case, we will launch two distinct recursive searches. The first one on the left child that will add all the transcripts contained in the subtree of a right child upon descending on the left one while the other search will be performed on the right child that will add all the transcripts contained in the subtrees of a left child upon descending on the right one. This procedure, described in Berg[20] as vsplit(), ensures that any gene overlapping the genomic window formed by adding a range parameter on each side of the coordinate will be reported. We currently limit the range to a maximum of 100 Mbp. However, this can be easily modified in the source code if need be.

After the overlapping splicing isoforms or genomic features are returned, we cluster the isoforms according to which genes they belong to. Segtor reports the results in terms of which input queries are either upstream, downstream or within intronic or exonic regions. In the case of SNVs, we determine which ones fall inside exonic regions, whether they are contained within untranslated regions (5′/3′ UTR) or within coding regions and report the amino acid change if any. It also reports the ones that landed within two base pairs of a donor or acceptor site inside intronic regions. In either case, the raw results as well as statistics tallying the number of input queries that fell within various portions of genes are reported.

For the detection of the closest TSS, Segtor builds a sorted array of all the TSSs of the genes in the database and performs a binary search to locate the closest TSS. It reports the distances to the closest TSSs into bins to readily produce a bar chart.

### Through the online interface

**Figure 3:**
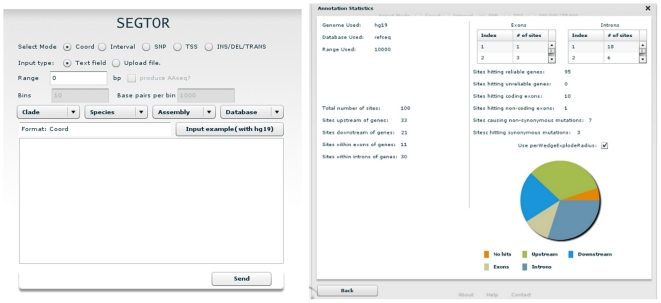
Screenshots of Segtor's online interface, used for handling small queries for test purposes. The figure to the left shows the initial interface with the various modes and the option of either entering coordinates through the text field for small queries or by uploading a file. The figure to the right shows examples of statistics produced by the annotation of SNVs showing how many SNVs landed in which part of genes and how many were classified as either synonymous or non-synonymous for those landing in coding portions of genes.

An interface in Adobe Flex (see figure 3) was designed to allow outside users to submit small queries for test purposes. The system does not require any login or email address and most requests are answered within 10 seconds. Once a request is done processing, the user is offered 2 outputs: the annotation for each input and a tally which includes a histogram for closest TSS detection and pie charts for coordinates, intervals and SNVs.

## Results

### Speed

#### Comparison against a DBMS

**Figure 4:**
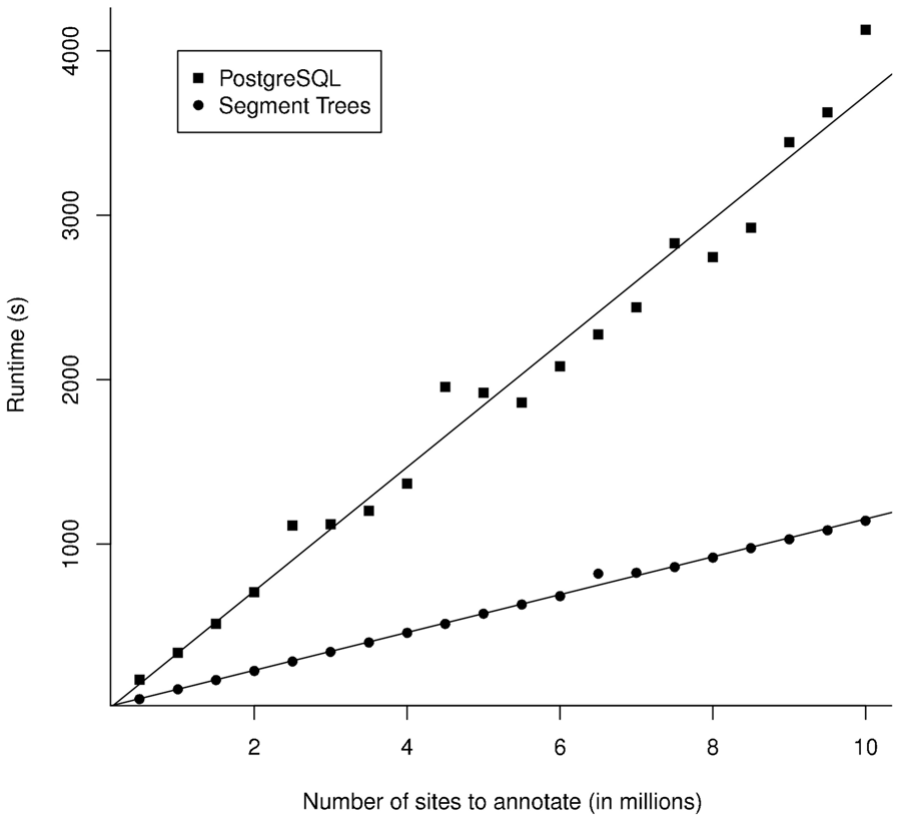
Comparison of the time required by our segment tree structure implementation versus a DBMS for the retrieval of genes that overlap a certain number of genomic coordinates. As DBMS are usually the method of choice for storing genomic annotation, a comparison between the time needed by our data structure and a optimized PostgreSQL table structure was needed to assess which solution was practically faster. Hence, in addition to being easier to use for end-users, forgoing the use of a DBMS for genomic annotation is also faster.

As we alluded to earlier, groups traditionally solve the annotation problem by resorting to storing and querying the data using a local DBMS. Hence, we compared the efficiency of a segment tree loaded in memory in finding the genes overlapping a set of genomic coordinates that were randomly generated on the hg18 version of the human genome against queries to a PostgreSQL server installed on the local machine. The SQL tables were optimized to guarantee rapid retrieval of the genes using SQL indices built on the coordinate fields. We loaded and queried the segment tree data structure and executed the SQL select statements using a Perl script. Our data structure is about 4 times faster in finding the appropriate genes in the database (see figure 4). The segment tree structure built using the RefSeq database from the UCSC Genome Browser downloaded on 04/27/2010 with 34,418 transcripts had a memory footprint of 0.5 GB and required 72 MB of space on the hard drive. The segment tree structure required 41 seconds to be built and was subsequently stored on the local disk. Retrieving it and loading it into memory required 2.6 seconds.

#### Against large datasets

We ran Segtor on a server with 132G of RAM using a single 2.4GHz CPU and Perl version 5.10.1 for a total of 10 minutes (600 seconds) to measure the annotation speed. This experiment was conducted on 3 different files stemming from an RNA-Seq alignment using coordinates (for the coordinate annotation and closest TSS mode), intervals (for the interval annotation mode) and SNVs (for the SNV annotation mode). Once loaded into memory, the index for the RefSeq database for the February 2009 build of the Human Genome (hg19) has a RAM footprint of about 1.5G. However, we recommend running Segtor on a server with a large RAM (32G-64G) when using dbSNP comparisons or using the very large human dbEST. Our results presented in table 1 show that Segtor is fast and suitable for use against large datasets produced by high-throughput sequencing.

**Table 1:** Amount of queries processed in 10 minutes.

Input type	# of inputs processed
Coordinates	3,671,706
Intervals	1,707,074
SNVs	3,034,284
Closest TSS	5,348,307

To evaluate Segtor's speed, we created 4 datasets and evaluated how many sites were annotated in 10 minutes (600 seconds) using a single CPU at 2.4 GHz. Perl version 5.10.1 was used as our interpreter. This speed makes Segtor a well-suited tools to routinely annotate large sets stemming from NGS runs.

### Accuracy

#### Against an annotated dataset

To assess the accuracy of our results in annotating known SNPs, we downloaded an annotated set of 74,713 missense mutations from the Catalogue of Somatic Mutations in Cancer (COSMIC) database[21] to determine whether the annotation provided by Segtor would be equivalent to the original one (Data S1). Aside from a few gene symbol aliases, the existing annotation and impact on protein sequence described in the COSMIC database were identical to those found in Segtor's output. For example, the subset of 51 non-synonymous mutations described for the HCC1954 breast cancer cell line[22] were correctly re-annotated by Segtor and the amino acid change can be summarized as follows: 9 polar to nonpolar, 15 nonpolar to polar and 27 with no influence in the amino acid sidechain hydrophobicity.

### NGS case studies

**Figure 5:**
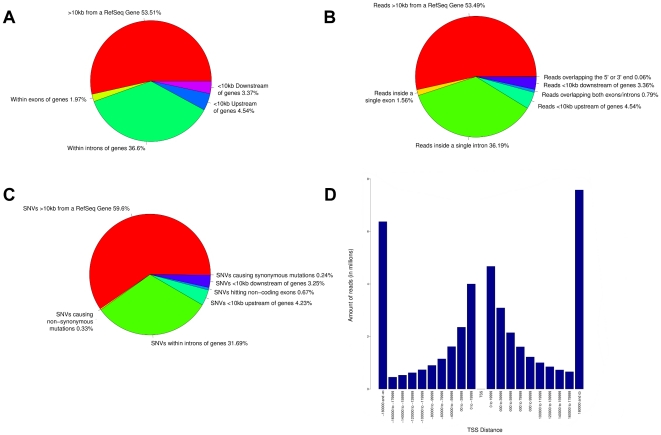
Example of statistics that can be obtained by running Segtor on 42,652,991 uniquely aligned Illumina reads and 2,707,221 putative SNVs. This graph represents the annotation modes for which statistics are available namely, annotation of a) coordinates, b) intervals, c) SNVs and d) closest TSS. A range of 10kb was used for our definition of upstream/downstream.

To show Segtor's effectiveness on NGS data, we downloaded a set of paired-end Illumina reads from SRX016474 dataset from NCBI's Sequence Read Archive (SRA[23]) and aligned them to the February 2009 build of the Human Genome (hg19) using novoalign[24]. We called putative SNVs using SNVMix. We converted the resulting BAM file and the putative SNVs to the native format of Segtor using the aforementioned conversion script. We extracted the 5′ coordinate and the interval produced by each read from the 42,652,991 aligned reads and kept all of the 2,707,221 putative SNVs called by SNVMix regardless of quality. We annotated all 3 sets against RefSeqs to determine the relative position and closest TSS of the 5′ ends, the relative position of the intervals created by reads and finally, the relative position of SNVs and their classification into synonymous and non-synonymous. Pie charts and histograms were created using R[25] and the results are presented in figure 5. We were also successful in annotating insertions/deletions detected by VarScan (data not shown).

**Figure 6:**
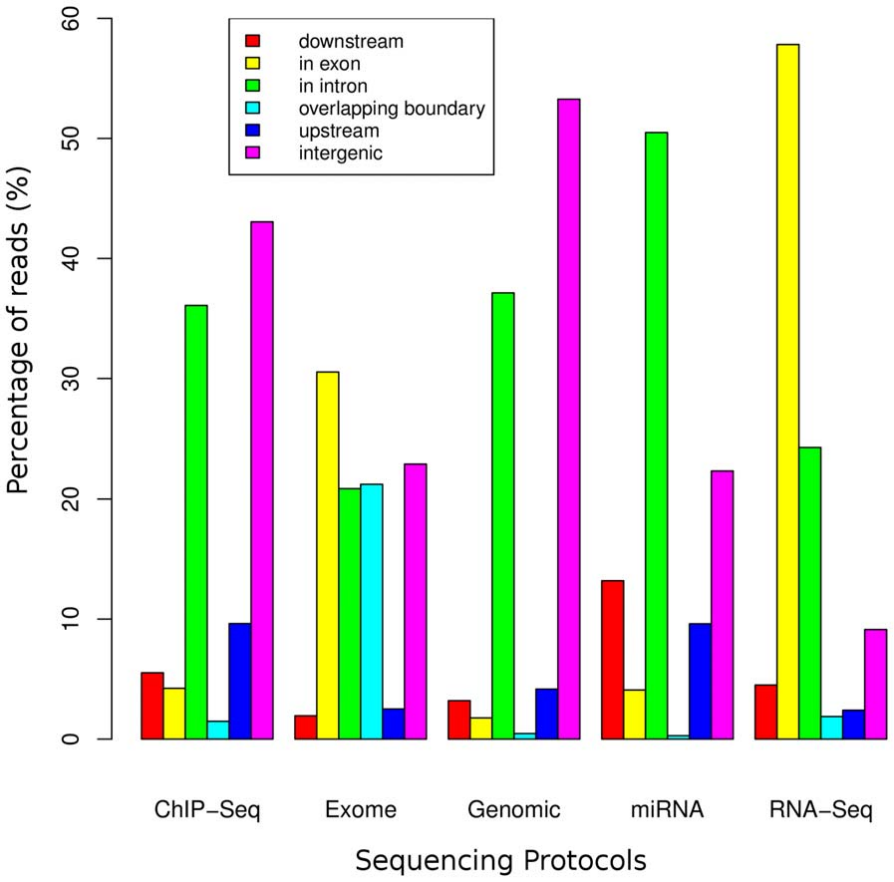
The distribution of the reads from various sets which use different sequencing protocols. The following sets ERX004477, SRX005927, SRX000644, SRX010851 and SRX017222 are Illumina runs which came respectively from a ChIP-Seq, exome capture, genomic sequencing, microRNA discovery and RNA-Seq protocol. They were retrieved from the SRA, aligned to the human genome and their resulting reads were annotated using Segtor with the RefSeq database. The genomic context of the reads varies greatly according to the sequencing protocol that was used. We used a range of 10kb on either side of every gene.

To measure whether that distribution would vary should a different experimental protocol be used, we retrieved from the SRA and aligned to the human genome a set from the following experiments: ERX004477, SRX005927, SRX000644, SRX010851 and SRX017222 which respectively use a ChIP-Seq, exome capture, genomic sequencing, microRNA discovery and RNA-Seq. The sets stem from Illumina sequencers and were aligned using novoalign. The different distribution of the reads on a genome-wide basis on presented in figure 6. The distribution for the ChIP-Seq protocol is consistent with previously reported enrichment values for various regions of the genome[26].

**Figure 7:**
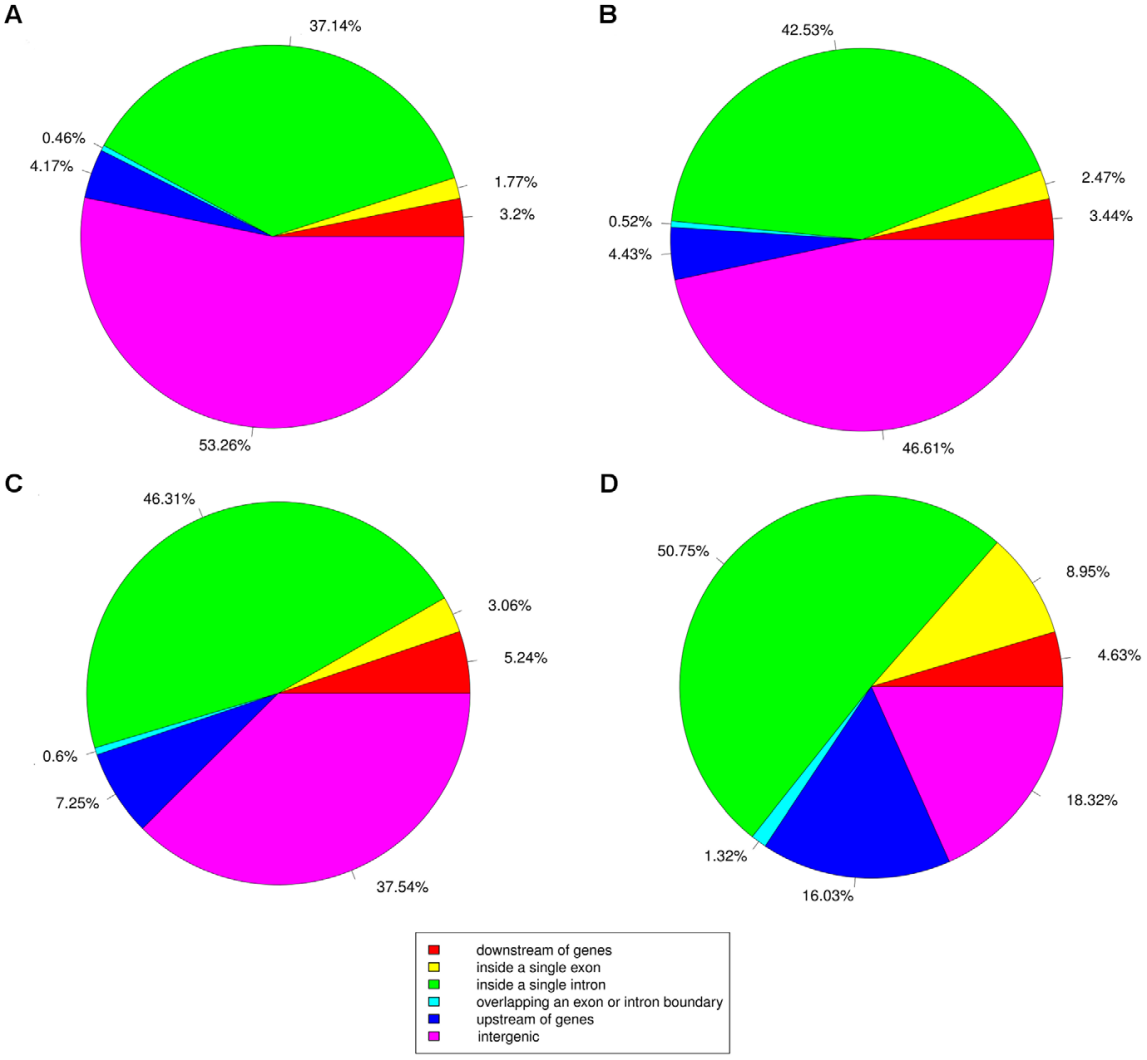
The distribution of the reads when using different databases as source of annotation. The same set presented in figure 5 was re-annotated using the latest version of a) RefSeq genes, b) UCSC Known Genes, c) Ensembl Genes and d) dbEST. Whether reads are labeled as intergenic or within genes varies according to the database that was used. Stricter databases like RefSeq will result in a greater number being labeled as intergenic compared to a more inclusive databases like dbEST. Again, a range of 10kb on either side of every gene was used.

To evaluate whether the definition of intergenic would vary according to the choice of the database, we downloaded the latest database files and re-annotated the run stemming from SRX000644 using the RefSeq, Ensembl Genes, UCSC Known Genes and dbEST databases (figure 7). While going from a more conservative database like RefSeq to a more inclusive one like dbEST, the number of reads being labeled as intergenic decreased while the number of reads labeled as landing within genes increased.

## Discussion

### Comparison to existing tools

The functionalities of Segtor can be dichotomized into annotating coordinates or intervals and annotating detected mutations, namely SNVs, indels and translocations.

The first task of detecting the overlap between genomic coordinates or small intervals and annotated genes can be readily accomplished on the UCSC Genome Browser via the Table Browser[27] for very small queries set but rapidly becomes impractical for large amounts of data. Larger sets could be analyzed using either the Galaxy server[28] or locally, using either BEDtools[29] or Tabix[30] on downloaded gene tables. This approach, however, requires substantial bioinformatics support to do pre- and post-processing to gather annotation statistics and is not suitable for smaller labs with limited access to programmers. Certain online tools like GPAT[31] allow annotation of a certain number of coordinates through an online interface but cannot be easily run locally and is limited to only a few species. CisGenome[32] and PeakAnalyzer[33] also allow the possibility of annotating genomic coordinates but have been mostly aimed at ChIP-on-chip and ChIP-Seq data. A comparison between the features of our software and the aforementioned tools is provided in Table 2.

**Table 2:** Comparison chart for various coordinate/interval annotation software.

	CisGenome	PeakAnalyzer	Galaxy	BEDtools	Segtor
Input type	mostly aimed at ChIP-seq coordinates	intervals	intervals	coordinates or intervals	coordinates or intervals
Platform	GUI	GUI and command line	web	command line	web and command line
Features	closest and neighboring	closest gene + closest TSS	find closest and overlap	find closest and overlap at a specified range	closest TSS/overlap at multiple specified ranges
Target database	genes	genes	must be customized	must be customized	genes/can be customized
Supported Species	limited to a dozen	limited to Human and Mouse only	Unlimited	Unlimited	Unlimited
Statistics	not readily	not readily	yes	not readily	yes

A comparison chart for available software/platforms for coordinates/intervals annotation.

As for the latter task of annotating mutations, webtools like SIFT[34], XVAR[35], SNPnexus[36] and the Genome Variation Server[37] can characterize known and novel SNPs but cannot be easily run locally against larger sets and against various species. A tool designed to be executed locally and able to suit the annotation needs of the resequencing community is be the Ensembl Variation API[38] which allows SNV analysis through their object oriented Perl API. However, Perl APIs require a Perl wrapper and queries to remote databases often hinder performance. Another tool called GAMES[39] identifies and annotates SNVs and indels in NGS projects but is currently limited to a single version of the human genome. Similar tools which can offer an improved annotation speed are SeqAnt[40] and AnnoVar[41] which aim at annotating SNVs and indels but provide little annotation information and do not produce any tally. Another software allowing the identification of the genomic context of SNV is SequenceVariantAnalyzer[42] which offers the possibility of having statistics regarding the genomic distribution of the SNVs or indels but like the previously mentioned tools, obtaining the entire mutated protein sequence for proteomic studies would involve substantial post-processing. We present a comparison of the characteristics of Segtor and the software that has been cited in table 3.

**Table 3:** Comparison chart for available variation annotation software.

	SeqAnt	Annovar	Sequence Variant Analyzer	Segtor
Input type	SNV/indels	SNV/indels	SNV/indels and structural variations	SNV/indels and translocations
Platform	web and command line	command line	GUI/command line	web and command line
Statistics	no	no	yes	yes
Supported Species	Limited	Unlimited	Human only	Unlimited
Amount of information	Limited	Limited	Extensive	Extensive
dbSNP comparison	yes	yes	yes	yes

A comparison chart for available software/platforms for SNV/indel annotation.

#### Comparison of current tools in annotating SNVs and coordinates

To further compare the performance and memory usage of Segtor against currently available tools which offer similar functionalities, we took the same 2,707,221 SNVs from the SRX016474 set described in the results section and used Segtor, SeqAnt, AnnoVar and SequenceVariantAnalyzer to annotate them. To evaluate the efficiency of software aimed at annotating coordinates, we used the 2,707,221 genomic coordinates of the SNVs as input for Segtor, CisGenome, PeakAnalyzer. Both SNVs and coordinate analyses are presented in table 4. Segtor offers comparable or better running time than available tools while having many novel features compared to software described in the literature. Regarding memory usage, Segtor is suitable to be used on a regular desktop computer.

**Table 4:** Features of the available tools for the analysis of coordinate and SNVs.

Software	Time	Peak Memory Usage (MB)	Comments
**For Coordinates**			
CisGenome	12m42s	11.2	• Can select different ranges to consider for upstream and downstream• Single output, no statistics
PeakAnnotator	unknown	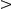 33000	The program exceeded the available RAM on our server
Segtor	5m48s	842	• Multiple fixed ranges for upstream and downstream• Various files depending on the biological question
**For SNVs**			
SeqAnt	63m11s	805	• Cannot specify a range parameter for upstream/downstream• Limited number of species
Annovar	3m18s	228	• Fast and memory efficient• Does not provide statistics, reports a single isoform per hit
Sequence Variant Analyzer	120m50s	7700	• Graphical User Interface• Provides greater information at the expense of speed
Segtor	8m58s	1579	• Produces output files and statistics on a per hit, per gene or per isoform basis• Can produce the set of mutated protein sequences

A case study of using the currently available software tools for annotating SNVs and coordinates to characterize genomic position of the 2,707,221 SNVs from the SRX016474 dataset. The corresponding coordinates of the SNVs were used as inputs for the software aimed at annotating coordinates. With the exception of Sequence Variant Analyzer which came with its own pre-compiled set of various gene databases, every tool in the list used RefSeq as source of annotation. These tests were conducted on an server with 8 CPUs at 2.5 GHz and 33 GB of RAM.

### Conclusion

As sequencing platforms are now releasing small versions aimed at individual laboratories at a more affordable price and, with the advent of personalized medicine, NGS platforms are poised to become ubiquitous. As the availability of programmers trained in bioinformatics has not always accompanied the rise of NGS platforms, easy-to-use tools to analyze data which do not require intricate parsing are increasingly needed. To illustrate this, some of the analysis we performed could have been done using a conjunction of existing tools with substantial support from programmers. However, the availability of a single ad hoc tools like Segtor would have enabled a small research group with a few computationally-savvy biologists to perform them. Furthermore, Segtor can be readily embedded within a data analysis pipeline for personal genomics.

### Availability and future work

Segtor was developed on Linux and needs to be launched from a UNIX terminal. It only requires a recent Perl 5 interpreter with standard modules and a C compiler. The limit to the number of queries or genes is only bounded by the available RAM. It requires a broadband connection to the internet to download data from the UCSC FTP site (ftp://hgdownload.cse.ucsc.edu/goldenPath/) to build new indices. It is freely available for academic and non-profit use and can be downloaded from http://lbbc.inca.gov.br/segtor/.

The source code could be adapted to work with other sources of genomic data and future versions will include a more diverse set of UCSC tracks and from different data sources.

## Supporting Information

Data S1 **Annotation of the 74,713 missense mutations from the COSMIC database using Segtor.** To verify whether the annotation provided by Segtor would be consistent with a pre-annotated set of SNVs, the set of 74,713 missense mutations from the COSMIC database was used as input for Segtor. The original annotation (gene name, isoform, original and mutant amino acid) was kept within the input ID (leftmost column). Leaving aside a few gene aliases, the annotation provided by Segtor was consistent with the original one.
(OUT)

## References

[1] Bentley DR (2006). Whole-genome re-sequencing.. Curr Opin Genet Dev.

[2] Hawkins RD, Hon GC, Ren B (2010). Next-generation genomics: an integrative approach.. Nat Rev Genet.

[3] Danecek P, Auton A, Abecasis G, Albers CA, Banks E (2011). The variant call format and VCFtools.. Bioinformatics.

[4] Pakakasama S, Kajanachumpol S, Kanjanapongkul S, Sirachainan N, Meekaewkunchorn A (2008). Simple multiplex RT-PCR for identifying common fusion transcripts in childhood acute leukemia.. Int J Lab Hematol.

[5] Bentley DR, Balasubramanian S, Swerdlow HP, Smith GP, Milton J (2008). Accurate whole human genome sequencing using reversible terminator chemistry.. Nature.

[6] Varas F, Stadtfeld M, de Andres-Aguayo L, Maherali N, di Tullio A (2009). Fibroblast-derived induced pluripotent stem cells show no common retroviral vector insertions.. Stem Cells.

[7] Alekseyenko AV, Lee CJ (2007). Nested Containment List (NCList): a new algorithm for accelerating interval query of genome alignment and interval databases.. Bioinformatics.

[8] Werner T (2010). Next generation sequencing in functional genomics.. Brief Bioinformatics.

[9] Bentley J (1977). Solutions to Klees rectangle problems.. Unpublished manuscript, Dept of Comp Sci.

[10] Pruitt KD, Tatusova T, Klimke W, Maglott DR (2009). NCBI Reference Sequences: current status, policy and new initiatives.. Nucleic Acids Res.

[11] Hubbard T, Barker D, Birney E, Cameron G, Chen Y (2002). The Ensembl genome database project.. Nucleic Acids Res.

[12] Hsu F, Kent WJ, Clawson H, Kuhn RM, Diekhans M (2006). The UCSC Known Genes.. Bioinformatics.

[13] Boguski MS, Lowe TM, Tolstoshev CM (1993). dbEST–database for “expressed sequence tags”.. Nat Genet.

[14] Sherry ST, Ward MH, Kholodov M, Baker J, Phan L (2001). dbSNP: the NCBI database of genetic variation.. Nucleic Acids Res.

[15] Desmet FO, Hamroun D, Lalande M, Collod-Beroud G, Claustres M (2009). Human Splicing Finder: an online bioinformatics tool to predict splicing signals.. Nucleic Acids Res.

[16] Woolfe A, Mullikin JC, Elnitski L (2010). Genomic features defining exonic variants that modulate splicing.. Genome Biol.

[17] Li H, Handsaker B, Wysoker A, Fennell T, Ruan J (2009). The Sequence Alignment/Map format and SAMtools.. Bioinformatics.

[18] Goya R, Sun MG, Morin RD, Leung G, Ha G (2010). SNVMix: predicting single nucleotide variants from next-generation sequencing of tumors.. Bioinformatics.

[19] Koboldt DC, Chen K, Wylie T, Larson DE, McLellan MD (2009). VarScan: variant detection in massively parallel sequencing of individual and pooled samples.. Bioinformatics.

[20] Berg M (2008). Computational geometry: algorithms and applications..

[21] Forbes SA, Bhamra G, Bamford S, Dawson E, Kok C (2008). The Catalogue of Somatic Mutations in Cancer (COSMIC).. Curr Protoc Hum Genet.

[22] Gazdar AF, Kurvari V, Virmani A, Gollahon L, Sakaguchi M (1998). Characterization of paired tumor and non-tumor cell lines established from patients with breast cancer.. Int J Cancer.

[23] Sequence Read Arquive http://www.ncbi.nlm.nih.gov/Traces/sra.

[24] Novoalign. URL http://www.novocraft.com/

[25] R Development Core Team (2009). R: A Language and Environment for Statistical Computing. R Foundation for Statistical Computing, Vienna, Austria.. http://www.R-project.org.

[26] Schmidt D, Schwalie PC, Ross-Innes CS, Hurtado A, Brown GD (2010). A CTCF-independent role for cohesin in tissue-specific transcription.. Genome Res.

[27] Rhead B, Karolchik D, Kuhn RM, Hinrichs AS, Zweig AS (2010). The UCSC Genome Browser database: update 2010.. Nucleic Acids Res.

[28] Goecks J, Nekrutenko A, Taylor J, Afgan E, Ananda G (2010). Galaxy: a comprehensive approach for supporting accessible, reproducible, and transparent computational research in the life sciences.. Genome Biol.

[29] Quinlan AR, Hall IM (2010). BEDTools: a flexible suite of utilities for comparing genomic features.. Bioinformatics.

[30] Li H (2011). Tabix: fast retrieval of sequence features from generic TAB-delimited files.. Bioinformatics.

[31] Krebs A, Frontini M, Tora L (2008). GPAT: retrieval of genomic annotation from large genomic position datasets.. BMC Bioinformatics.

[32] Ji H, Jiang H, Ma W, Johnson DS, Myers RM (2008). An integrated software system for analyzing ChIP-chip and ChIP-seq data.. Nat Biotechnol.

[33] Salmon-Divon M, Dvinge H, Tammoja K, Bertone P (2010). Peakanalyzer: Genome-wide annotation of chromatin binding and modification loci.. BMC Bioinformatics.

[34] Ng PC, Henikoff S (2001). Predicting deleterious amino acid substitutions.. Genome Res.

[35] Reva B, Antipin Y, Sander C (2007). Determinants of protein function revealed by combinatorial entropy optimization.. Genome Biol.

[36] Chelala C, Khan A, Lemoine NR (2009). SNPnexus: a web database for functional annotation of newly discovered and public domain single nucleotide polymorphisms.. Bioinformatics.

[37] The Genome Variation Server http://gvs.gs.washington.edu/.

[38] Rios D, McLaren WM, Chen Y, Birney E, Stabenau A (2010). A database and API for variation, dense genotyping and resequencing data.. BMC Bioinformatics.

[39] Sana ME, Iascone M, Marchetti D, Palatini J, Galasso M (2011). GAMES identifies and annotates mutations in next-generation sequencing projects.. Bioinformatics.

[40] Shetty A, Athri P, Mondal K, Horner V, Steinberg K (2010). Seqant: A web service to rapidly identify and annotate dna sequence variations.. BMC Bioinformatics.

[41] Wang K, Li M, Hakonarson H (2010). ANNOVAR: functional annotation of genetic variants from high-throughput sequencing data.. Nucleic Acids Res.

[42] Ge D, Ruzzo EK, Shianna KV, He M, Pelak K (2011). SVA: software for annotating and visualizing sequenced human genomes.. Bioinformatics.

